# Survival of a case of *Bacillus cereus* meningitis with brain abscess presenting as immune reconstitution syndrome after febrile neutropenia – a case report and literature review-

**DOI:** 10.1186/s12879-019-4753-1

**Published:** 2020-01-06

**Authors:** Yusuke Koizumi, Takafumi Okuno, Hitoshi Minamiguchi, Keiko Hodohara, Hiroshige Mikamo, Akira Andoh

**Affiliations:** 1 Department of Clinical Infectious Diseases, Aichi Medical University Hospital, 1-1 Yazakokarimata, Nagakute, Aichi 480-1195 Japan; 2 Department of Hematology, Shiga University of Medical Science, Otsu, Shiga Japan

**Keywords:** *Bacillus cereus*, Meningitis, Bacteremia, Febrile neutropenia, Immune reconstitution syndrome

## Abstract

**Background:**

*Bacillus cereus* sometimes causes central nervous system infection, especially in compromised hosts. In cases of meningitis arising during neutropenia, CSF abnormalities tend to be subtle and can be easily overlooked, and mortality rate is high. We report a survived case of *B. cereus* meningitis/brain abscess in severe neutropenia, presenting as immune reconstitution syndrome.

**Case presentation:**

A 54-year-old Japanese female with acute myelogenous leukemia developed *B. cereus* bacteremia and meningitis during consolidation chemotherapy. At the onset, she presented with mild meningism. She had marked leukocytopenia (WBC <100/μL, neutrophils 0/μL) and lumbar puncture yielded only mild pleocytosis. She was transferred to intensive care unit, and meropenem, linezolid and vancomycin was started. With intensive therapy, she recovered and once became afebrile. On day 19, however, her fever, meningism and consciousness level dramatically worsened despite recovery of bone marrow function. The antimicrobial chemotherapy was continued and finally she was cured with no complications.

**Conclusions:**

With early diagnosis and prompt initiation and of antibiotics, the case was successfully treated without any sequelae. It is important to remember that, even under optimal antimicrobial therapy, bone marrow recovery can cause transient reaggravation of the disease. In such cases, timely and appropriate evaluation should be done to make the clinical decision to change, continue, or intensify treatment.

## Background

*Bacillus cereus* is a Gram positive, aerobic facultative, spore-forming, rod-shaped bacterium. It is motile, catalase positive and ubiquitous, found in air, soil, water, and food [1]. Because of the ubiquitous distribution of *B. cereus* in food products, the bacterium is ingested in small numbers and becomes part of the transitory human intestinal flora [2]. Traditionally, the detection of *Bacillus* species in sterile samples has been regarded as contamination. However, the organism has the potential to cause severe infectious diseases in immunocompromised hosts [1]. Herein, we report a case of *B. cereus* meningitis with brain abscess, arising in the setting of neutropenia, and presenting as immune reconstitution syndrome after bone marrow recovery.

## Case presentation

A 54-year-old Japanese female was diagnosed with acute myeloid leukemia (AML). Complete remission was achieved after induction chemotherapy, but 14 months later the disease relapsed. During the re-induction chemotherapy (FLAGM; fludarabine, cytarabine, granulocyte colony stimulating factor, and mitoxantrone), she experienced severe septic shock caused by *Klebsiellla pneumoniae* bacteremia requiring intensive care. During the consolidation therapy (high dose cytarabine), oral polymyxin B, sulfamethoxazole/trimethoprim, voriconazole, and acyclovir were administered as prophylaxis.

On day 18 of consolidation chemotherapy (day 1 of infection), she complained of fever and chills. Meropenem 3 g/day was started after blood culture sampling. On day 2, headache and nausea appeared. Diarrhea was absent, and the vital signs were as follows: blood pressure 124/67 mmHg, heart rate 86 bpm, and body temperature 39.5 °C. At this time, she was conscious and lucid. Neurologically, no abnormal focal signs, including nuchal rigidity, were noted. Laboratory tests showed marked leukocytopenia (WBC < 100/μL, neutrophils 0/μL) with slight C-reactive protein elevation (1.32 mg/dL). The blood culture detected a Gram-positive rod-shaped bacterium on day 2, and the pathogen was identified as *B. cereus* on day 4. The strain was resistant to penicillin and quinolones, but sensitive to carbapenems, vancomycin, and linezolid.

On day 3, disorientation, slurred speech, and confusion appeared. Slight nuchal rigidity was observed, and the neck flexion test was positive. Although meningitis was suggested, lumbar puncture was not performed because of severe thrombocytopenia (21,000/μL). Instead, linezolid (1200 mg/day), vancomycin (targeted trough of 20 mg/dL), and acyclovir (30 mg/kg/day) were administered in addition to high dose meropenem (6 g/day).

On day 5, she was transferred to the intensive care unit because of shock. At this time, lumbar puncture yielded the following results: opening pressure: 18 cmH_2_O; cells: 43/mm^3^ (mononuclear cells dominant, with no abnormal cells); glucose: 31 mg/dL; protein: 155 mg/dL; negative for HSV-DNA, VZV-DNA, CMV-DNA, HHV-6 DNA, Cryptococcus-Antigen and WT-1 (Wilms Tumor-1). Cerebrospinal fluid (CSF) culture was negative, but *B. cereus* PCR was positive, leading to the diagnosis as *B. cereus* meningitis.

With inotropic agents, deep sedation, and antimicrobials, she recovered and became afebrile. On day 19, however, her fever, meningism and consciousness level dramatically worsened despite recovery of bone marrow function. The CSF findings revealed markedly increased cells (2040 cells/mm^3^), and decreased glucose level (12 mg/dL). The CSF culture and PCR for *B. cereus* were both negative, and other diseases were excluded by the aforementioned examinations. Therefore, in concert with the increased leukocytes in peripheral blood and CSF, it was suggestive of immune reconstitution syndrome after *B. cereus* meningitis. The gadolinium enhanced magnetic resonance imaging (MRI) performed on day 15 had already detected meningeal thickening and several ring enhancement lesions. Glucocorticoid administration was not used to treat immune reconstitution syndrome. Meropenem, vancomycin, and linezolid were continued for 26, 40, and 45 days, respectively. They were stopped after CSF and MRI findings were confirmed to be normalized. On day 90, she was discharged to her home without any sequela, and her AML remains in remission for 5 years.

## Discussion and conclusions

Central nervous system infections caused by *B. cereus* can be classified as three diseases: 1) postsurgical meningitis, ventriculitis or brain abscess, which can occur several days after a surgical procedure and often affects devices [3]; 2) neonatal meningitis or encephalitis occurring in premature neonates at 7 days of age (median, range 1–49 days) [4]; 3) necrotizing meningitis or hemorrhage in cases of severe neutropenia, predominantly affecting patients with leukemia (Table 1) [3–14].

**Table 1:** Three Types of Central Nervous System *Bacillus cereus* Infection

	Post-Surgical Procedure/Device Infection	Neonatal Infection	Severe Neutropenia
Risk Population	Ommaya reservoir	Premature birth: 32 (27–36) weeks	WBC count <100/uL
	Post intrathecal injection	Low body weight infant:1500 (830–3760) g	Induction therapy against AML
Disease Onset	Several days after procedures	7 (1–49) days after birth	Around 10 days of neutropenia
Mode of Infection	Meningitis	Meningoencephalitis	Necrotizing meningoencephalitis
	Ventriculitis		Cerebral hemorrhage
	Brain abscess		Subarachnoid hemorrhage
Clinical Course and Prognosis	Responds well to antimicrobial therapy	Progresses within hours	Progresses within hours
		Leads to death within days	Leads to death within days
Cerebrospinal Fluid Findings	Up to 10^5^ cells /mm^3^	Around 10^2–3^ cells /mm^3^	Around 10^2^ cells /mm^3^
Mortality rate	Low	75% (12/14 cases)	79% (11/14 cases)
References	3	3, 4	3, 6–14

While postsurgical infections typically resolve in spite of marked CSF pleocytosis, in the order of 10^5^ cells/mm^3^, the latter two infection types have very poor prognoses with mortality rate greater than 75%. Notably, in many cases of these two malicious diseases, CSF cell counts are usually less than 10^2^ cells/mm^3^.

Fourteen cases of neutropenia-associated *B. cereus* meningitis have been reported so far (Table 2). All but one case had hematological malignancies (AML: 6, acute lymphoblastic leukemia: 7, myelodysplastic syndrome: 1, aplastic anemia: 1) and all had severe neutropenia of less than 500/μL at the onset of disease. Moreover, 79% had less than 100/μL blood leukocytes, suggesting a role for severe neutropenia in the pathogenesis of this disease. The cases demonstrated mild or absent CSF pleocytosis, which is considered to be a result of both the severely immunodeficient state of the patient and the character of *B. cereus* infection in itself, which causes little inflammatory reaction [15].

**Table 2:** Clinical Characteristics of Neutropenia-Related *Bacillus cereus* Meningitis

Symptoms at Onset														
Case	Year	Age	Underlying Diseases	WBC Count at Onset	Fever	Headache	Vomiting	Diarrhea	Abdominal Pain	Neurological Abnormalities	CNS Disease	Treatment	Outcome	Reference
1	1973	63	AML	400^a^	+					Coma	Brain abscess	GM, OXA, CBC	Dead	5
2	1981	19	AML	<100	+						Meningitis	GM, PCG	Dead	6
3	1988	67	MDS/AML	100^a^	+	+	+				Meningoencephalitis/SAH	GM, LM, PIPC	Dead	7
4	1989	3	ALL	20	+					Lethargy	Brain abscess	CP, VCM, GM, RFP	Alive	8
5	1995	26	AML	20			+			Visual disturbance	Meningoencephalitis/SAH	CAZ	Dead	9
6	1997	64	AML	300	+		+	+			Meningoencephalitis/SAH	PIPC, GM, CPZ, CTX, ABPC	Dead	10
7	1997	20	ALL	0	+		+	+	+	Speech disturbance, Sensory disturbance	Cerebral infarction (basal ganglia)		Dead	11
8	1999	13	ALL	0	+				+		Meningoencephalitis/hydrocephalus		Alive, Severe sequelae	11
9	1999	15	ALL	0		+			+		Meningoencephalitis		Dead	11
10	1999	30	AML	<100	+				+	Delirium, hyperventilation	Necrotic meningoencephalitis	CAZ, AMK	Dead	12
11	1999	43	AML	<100	+	+	+	+	+	Delirium, decerebrate rigidity	Meningoencephalitis	CAZ, AMK → VCM, GM & others	Dead	12
12	1999	14	ALL	<100	+					Delirium, epilepsy, coma	Meningoencephalitis	CAZ, AMK & others	Dead	12
13	2003	16	AA	90	+	+				Nuchal rigidity, epilepsy, consciousness disturbance	Meningoencephalitis	CAZ, IPM/CS	Dead	13
14	2005	19	HD	0	+					Confusion, epilepsy, hemiparesis	Meningoencephalitis	ABPC, AMK, CPFX, TEIC, CLDM	Alive	14
Our	case	54	AML	<100	+	+	+			disorientation, slurred speech, and confusion	Meningoencephalitis/hydrocephalus brain abscess	MEPM, LZD, VCM	Alive	

^a^denotes the number of neutrophils

Abbreviations: *AA* Aplastic anemia, *ABPC* Ampicillin, *ALL* Acute lymphoblastic leukemia, *AMK* Amikacin, *AML* Acute myelogenous leukemia, *CAZ* ceftazidime, CBC, carbenicillin, *CLDM* Clindamycin, *CNS* Central nervous system, *CP* Chloramphenicol, *CPFX* Ciprofloxacin, *CPZ* Cefoperazone, *CTX* Cefotaxime, *GM* Gentamicin, *HD* Hodgkin disease, *IPM/CS* Imipenem/cilastatin, *LM* Lincomycin, *LZD* Linezolid, *MDS* Myelodysplastic syndrome, *MEPM* Meropenem, *OXA* Oxacillin, *PCG* Penicillin G, *PIPC* Piperacillin, *RFP* Rifampin, *VCM* Vancomycin

Our case shared common features with previous reports such as severe neutropenia at the onset of meningitis and the complication of brain abscesses. While those are typical findings of neutropenia associated *B. cereus* meningitis, there are two particular features of this report. First, with an early diagnosis and administration of a contemporary antimicrobial regimen, we were able to cure the disease without any complications. The regimen contained antibiotics with good penetrance, including linezolid, high dose meropenem, and a well-controlled dose of vancomycin that achieved a serum trough level of 20 μg/mL. This well-structured regimen was started immediately after the onset of meningitis, though the physical findings were subtle in the early phase, and CSF findings at day 5 showed only a moderate level of pleocytosis. This early diagnosis is crucial for the rapid initiation of intensive antimicrobial therapy.

**Fig. 1:**
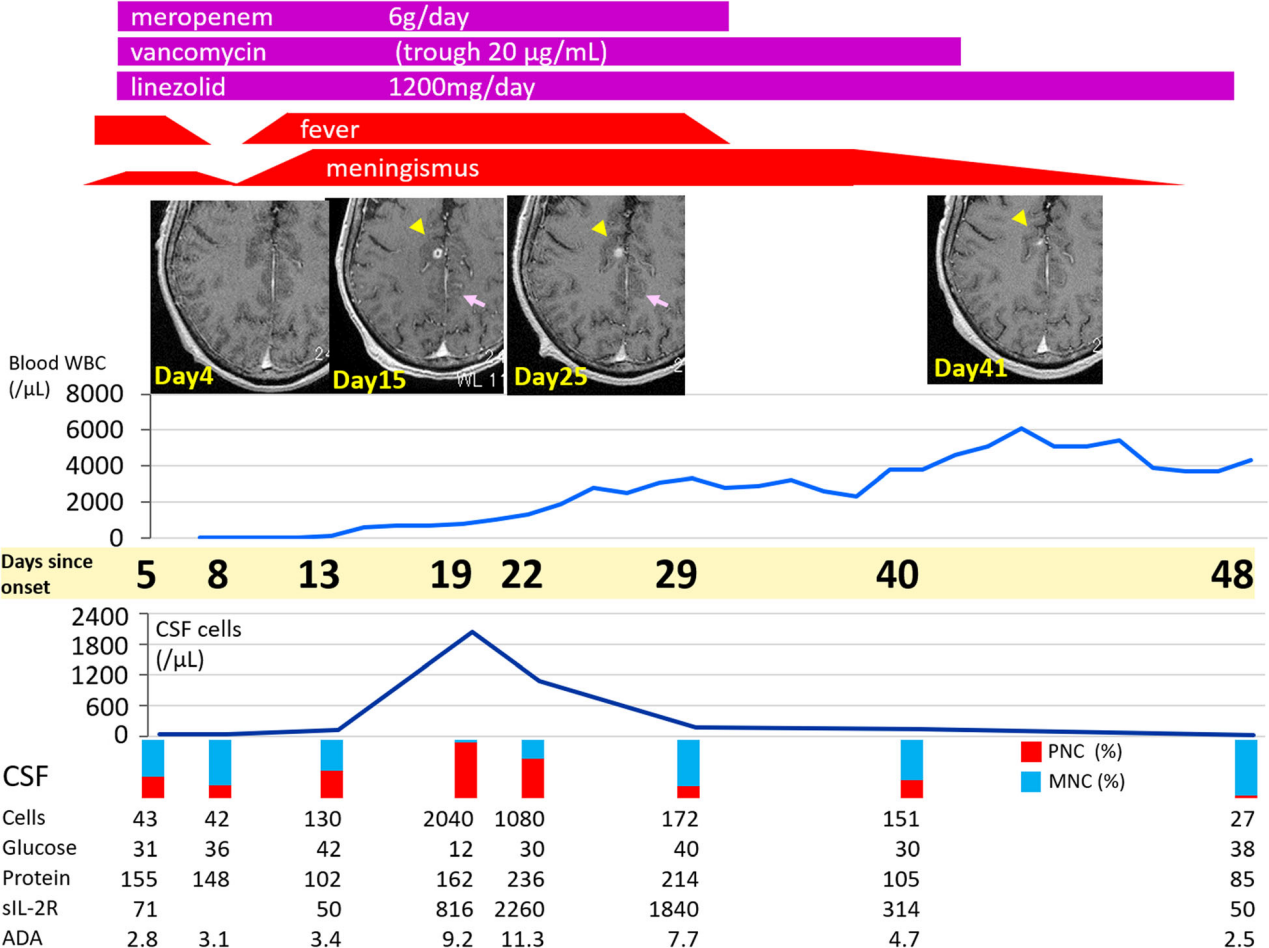
Clinical course. The figure indicates the clinical course of the patient from day 5 to day 48, when clinical symptoms resolved. The line plot shows the trend in blood leukocytes (above) and CSF cells (below). The vertical rectangle indicates the percentage of polymorphonuclear cells (red) in the CSF. On day 5, CSF showed moderate pleocytosis, with conspicuous protein increase. Antibiotic therapy was effective, leading to defervescence and neurological improvement. On day 13, however, fever and nuchal rigidity worsened. CSF findings showed 130 cells/μL with an increased proportion of polymorphonuclear cells. The MRI findings on day 15 revealed abscess lesions (arrow heads) and meningeal thickening (arrows). On day 19, meningism worsened remarkably, and CSF cells peaked at 2040 cells/μL with 98% polymorphonuclear cells. Other causes were ruled out, and meropenem, vancomycin, and linezolid were continued. Gradually, MRI and CSF findings improved and the patient was discharged without sequelae. Note that the inflammatory findings, such as CSF cells, sIL-2R, and ADA were dramatically enhanced in response to the recovery of blood leukocyte count. The reference ranges are; Blood leukocytes 3000-8000/μL, CSF cells 0–5/μL, CSF protein 10–40 mg/dL, CSF glucose 50–80 mg/dL, and CSF sIL-2R, <50 U/mL

Second, we were able to closely track the CSF and MRI findings with the recovery of hematopoiesis. The patient’s meningitis symptoms were improving until day 19, when neutrophils rapidly increased. With the recovery of neutrophil count, a dramatic worsening of fever, consciousness and meningism was demonstrated. This could be considered as a type of immune reconstitution syndrome, though the term originally denoted the recovery of cellular immunity from AIDS or other immunosuppressive states. As shown in Fig. 1, we serially observed parameters of cellular immunity as well as neutrophil count. Interestingly, the severity of meningitis symptoms clearly synchronized with the blood neutrophil count, and the correlation is even more dramatic for CSF cells, CSF soluble interleukin-2 receptor (sIL-2R), and CSF adenosine deaminase (ADA). On day 19, the vast majority of CSF cells were polymorphonuclear cells (neutrophils). Later, CSF, sIL-2R and ADA increased, indicating that a typical inflammation cascade was ongoing. As differential diagnosis, other infections or an intracranial recurrence of AML were considered, but negative culture, cytology and clinical course were indicative of *B. cereus* meningitis. Therefore, we continued the antibiotics and the symptoms gradually improved.

In conclusion, we experienced a case of *B. cereus* meningitis with brain abscess during severe neutropenia from which the patient survived. With serial measurement of inflammatory / immunological markers, our report described the phenomenon resembling immune reconstitution syndrome, and we also proved that contemporary antimicrobial regimens can cure this severe disease. In cases of meningitis arising during neutropenia, CSF abnormalities tend to be subtle and can be easily overlooked. Because immediate diagnosis is crucial for survival, clinicians should be able to carefully recognize the symptoms and interpret the clinical data in order to rapidly initiate intensive treatment. Furthermore, it is important to remember that bone marrow recovery can cause transient reaggravation of the disease, even under optimal antimicrobial therapy. In such cases, timely and appropriate evaluation should be done to make the clinical decision to change, continue, or intensify treatment.

## Data Availability

The authors are ready to provide the data in response to the need.

## Abbreviations

ADA: Adenosine deaminase
AIDS: Acquired immunodeficiency syndrome
AML: Acute myeloid leukemia
CMV: Cytomegalovirus
CSF: Cerebrospinal fluid
FLAGM: Fludarabine, cytarabine, glanulocyte colony stimulating factor, and mitoxantrone combined therapy
HHV-6: Human herpes virus-6
HSV: Herpes simplex virus
LZD: Linezolid
MEPM: Meropenem
MNC: Mononuclear cells
PNC: Polymorphonuclear cells
sIL-2R: Soluble interleukin-2 receptor
VCM: Vancomycin
VZV: Varicella zoster virus
WBC: White blood cells
